# The Interaction Between *Endo*polygalacturonase From *Fusarium Moniliforme* and PGIP from *Phaseolus Vulgaris* Studied by Surface Plasmon Resonance and Mass Spectrometry

**DOI:** 10.1002/cfg.113

**Published:** 2001-12

**Authors:** Benedetta Mattei, Felice Cervone, Peter Roepstorff

**Affiliations:** 1 Dipartimento di Biologia Vegetale Università di Roma 'La Sapienza' Piazzale Aldo Moro 5 Roma 00185 Italy; 2 Department of Biochemistry and Molecular Biology University of Southern Denmark Odense University Campusvej 55 Odense M DK 5230 Denmark

## Abstract

A combination of surface plasmon resonance (SPR) and matrix-assisted laser-desorptionionization- time-of-flight mass spectrometry (MALDI-TOF-MS) was used to study the interaction between *endo*polygalacturonase (PG) from *Fusarium moniliforme* and a polygalacturonase-inhibiting protein (PGIP) from *Phaseolus vulgaris.* PG hydrolyses the homogalacturonan of the plant cell wall and is considered an important pathogenicity factor of many fungi. PGIP is a specific inhibitor of fungal PGs and is thought to be involved in plant defence against phytopathogenic fungi. SPR was used either to study the effect of the PG glycosylation on the formation of the complex with PGIP, and as a sensitive affinity capture of an interacting peptide from a mixture of PG fragments obtained by limited proteolysis. Mass spectrometry allowed to characterise the interacting peptide eluted from the sensor surface.


**Comparative and Functional Genomics**
*Comp Funct Genom* 2001; **2**: 359–364.
**DOI:** 10.1002/cfg.113

**Conference Paper**

### The interaction between *endo*polygalacturonase from *Fusarium moniliforme* and PGIP from *Phaseolus vulgaris* studied by surface plasmon resonance and mass spectrometry.

A presentation for the ESF workshop 'Proteomics: Focus on protein interactions'

Benedetta Mattei[1]*, Felice Cervone[1] and Peter Roepstorff[2]
[1] *Dipartimento di Biologia Vegetale, Università di Roma 'La Sapienza', Piazzale Aldo Moro 5, 00185 Roma, Italy*
[2] *Department of Biochemistry and Molecular Biology, University of Southern Denmark, Odense University, Campusvej 55, DK 5230 Odense M, Denmark*

**Correspondence to:*
*Dipartimento di Biologia Vegetale, Università di Roma 'La Sapienza', Piazzale Aldo Moro 5, 00185 Roma, Italy*
*E-mail: benedetta.mattei@uniroma1.it*

Received: 4 September 2001
Accepted: 14 September 2001
Published online: 16 October 2001

### Abstract

**A combination of surface plasmon resonance (SPR) and matrix-assisted laser-desorption-ionization-time-of-flight mass spectrometry (MALDI-TOF-MS) was used to study the interaction between *endo*polygalacturonase (PG) from *Fusarium moniliforme* and a polygalacturonase-inhibiting protein (PGIP) from *Phaseolus vulgaris*. PG hydrolyses the homogalacturonan of the plant cell wall and is considered an important pathogenicity factor of many fungi. PGIP is a specific inhibitor of fungal PGs and is thought to be involved in plant defence against phytopathogenic fungi. SPR was used either to study the effect of the PG glycosylation on the formation of the complex with PGIP, and as a sensitive affinity capture of an interacting peptide from a mixture of PG fragments obtained by limited proteolysis. Mass spectrometry allowed to characterise the interacting peptide eluted from the sensor surface. Copyright © 2001 John Wiley & Sons, Ltd.**

**Keywords: Surface plasmon resonance; mass spectrometry; protein–protein interaction; *endo*polygalacturonase; *Fusarium moniliforme*; PGIP**

### Introduction

Surface plasmon resonance (SPR) biosensors are important and versatile tools for studying protein–protein interactions; they allow to measure interactions in real time and require very little material, which usually does not need any chemical modification. SPR technology coupled with mass spectrometry can be also used to identify and characterise proteins eluted from sensor surfaces (Sönksen *et al.*, 2000; Williams, 2000). Once a sample containing a mixture of possible ligands is passed over the sensor surface, and binding is detected by the SPR signal, the identification of interacting proteins at the femtomole level is made possible by the use of sensitive mass spectrometers and advanced database searching algorithms (Nelson *et al.*, 2000).

We are studying the interaction between the endopolygalacturonase (PG) from the phytopathogenic fungus *Fusarium moniliforme* with PGIP-2 of *Phaseolus vulgaris*. PGs catalyse the fragmentation and solubilisation of homogalacturonan in the plant cell wall and play an important role during pathogenesis. Polygalacturonase-inhibiting proteins (PGIPs), present in the cell wall of many plants, form specific complexes with fungal PGs and favour the accumulation of oligogalacturonides able to elicit plant defence responses (Cervone *et al.*, 1997). PGIPs belong to a super-family of leucine-rich repeat (LRR) proteins. In a previous study, we

Copyright © 2001 John Wiley & Sons, Ltd.

showed that *F. moniliforme* polygalacturonase and PGIP-2 specifically interact with high affinity ($K_D$ = 47 nM), and we demonstrated that the residues in the predicted $\beta$–strand/$\beta$–turn motif of PGIP are critical for its affinity and specificity for the PG ligands (Leckie *et al.*, 1999). The residues of PG involved in the interaction with PGIP are still unknown.

One of the best characterised members of the LRR superfamily is ribonuclease inhibitor (RI), that utilises a large set of interactions to achieve tight binding to different members of the RNase family (Kobe *et al.*, 1996; Papageorgiou *et al.*, 1997).

In analogy with what is known for RI complexes, it is likely that a network of contacts occur between PG and PGIPs. The aim of this study was to gain information on which domain of PG contains the PGIP interacting residues and whether the glycosylation of the PG molecule has any effect on the interaction.

### Materials and methods

### Protein purification

PGIP-2 was purified from *Nicotiana benthamiana* plants infected with PVX as previously described (Mattei *et al.*, 2001).

*Fm*PG expressed in yeast was prepared and purified as previously described (Caprari *et al.*, 1996).

### Surface plasmon resonance

The interaction between *Fm*PG and immobilised PGIP-2 was measured in real time by surface plasmon resonance using BIACORE X(TM) equipment (BIACORE AB, Uppsala, Sweden). Protein interaction analyses were performed on research grade BIACORE CM5 sensor chips. For the immobilisation of PGIP-2 the sensor chip was activated by injection of 35 µl of 1 : 1 mixture of N-ethyl-N′-(3-diethylaminopropyl)carbodiimide and N-hydroxysuccinimide at 5 µl/min flow rate. Running buffer used during the immobilisation procedure was HBS (10 mM Hepes, pH 7.4, 150 mM NaCl, 0.005% [v/v] surfactant P20 from BIACORE, in distilled water). 40 µl of bean PGIP-2 at 100 ng/µl in 10 mM sodium acetate, pH 4.5, were injected over the sensor chip, followed by 35 µl of 1 M ethanolamine hydrochloride to block the remaining ester groups. A total of 4000–5000 RU (Resonance Units, which are proportional to the mass of protein bound on the surface of the chip) of PGIP-2 were immobilised onto the sensor chip surfaces, corresponding to a density of 4–5 ng/mm$^2$. A second flow-cell of the sensor chip was treated in the same way, except that buffer was injected

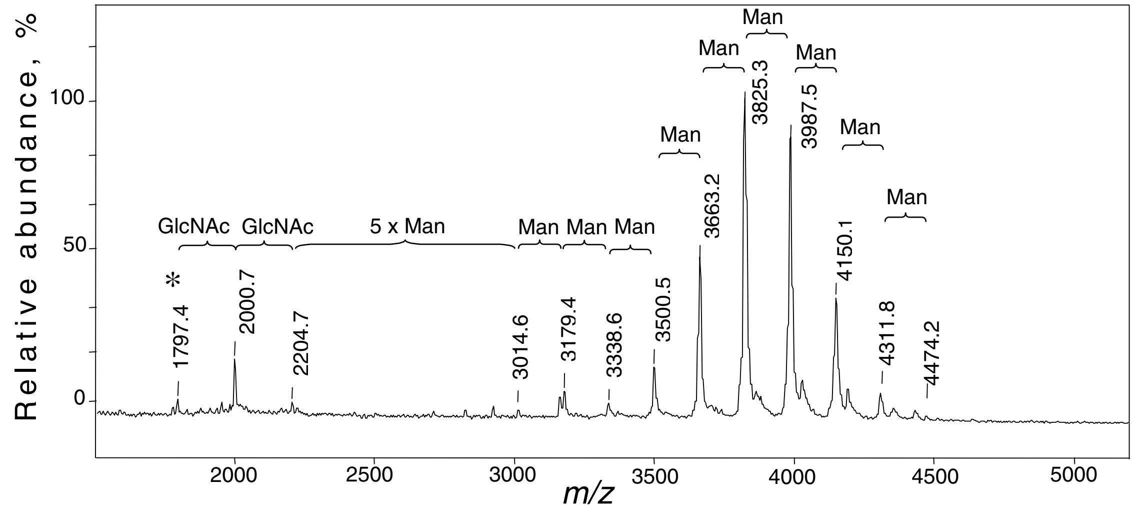

**Figure 1.** MALDI spectrum of a glycopeptide obtained by RP HPLC separation of the tryptic digest of *Fm*PG. The peak at *m/z* 1797.43 corresponds to the nonglycosylated form of the peptide spanning from amino acid 57 to amino acid 72, containing a potential *N*-glycosylation site at Asn65. Up to 14 Man residues are present in the glycan chain

Copyright © 2001 John Wiley & Sons, Ltd. 

instead of PGIP-2, and this surface was used as the reference flow-cell. In the interaction assays, PG solutions in 25 mM ammonium acetate buffer pH 5.0 were injected onto the PGIP-2 sensor chip at a flow rate of 5 µl/min.

Limited proteolysis of *Fm*PG was performed by incubating the native enzyme with Endoproteinase Lys C (Boehringer Mannheim GmbH, Frankfurt, Germany) using an enzyme/substrate ratio of 1 : 50 (w/w) in ammonium bicarbonate buffer pH 7.5 at 37°C; aliquotes taken at different times were analysed.

### Elution from the sensor chip

Elution from the sensor chip was performed essentially as described by Sönksen *et al.* (Sönksen *et al.*, 1998)with the following modifications: 10 µl of $NH_4HCO_3$ buffer pH 8 were used to elute most of the bound PG. The elution was performed at the beginning of the dissociation phase, without rinsing the system. The eluted sample was subjected to purification from salts and preparation for MALDI analysis by using reversed-phase nano-columns prepared as described by Gobom *et al.* (Gobom *et al.*, 1999).

### Mass spectrometry

MALDI mass spectra were acquired on a Voyager-Elite MALDI-TOF (Applied Biosystems, MA) mass spectrometer, operated in positive ion linear mode.

Matrices used were 2,5-dihydroxybenzoic acid (DHB) (Hewlett-Packard, Palo Alto,CA) 100 µL of 100 mM solution lyophilised and redissolved in an equal volume of 30% aqueous acetonitrile, 0.1% TFA and 4-hydroxy-α-cyanocinnaminic acid (HCCA) (Sigma, St. Louis, MO) 10 µg/µL dissolved in 70% aqueous acetonitrile, 0.1% TFA.

The spectra were externally calibrated by using HP peptide standard (oxytocin, arginine-8-vaso-pressin, angiotensin I, somatostatin, chicken atrial natriuretic peptide (ANP), human r insulin, r hirudin) (Hewlett-Packard, Palo Alto, CA).

### Results and discussion

PG from *F. moniliforme* (*Fm*PG) contains four potential *N*-linked glycosylation sites (Caprari *et al.*, 1993). The enzyme used for this study is *Fm*PG expressed in *Saccharomyces cerevisiae*. When analysed by SDS-PAGE, *Fm*PG showed three protein bands with molecular masses of 43, 46 and 50 kDa, respectively, corresponding to different glycoforms of the same polypeptide chain (Caprari *et al.*, 1996). The calculated molecular mass of the polypeptide is 36.2 kDa, indicating that the enzyme purified from yeast is heavily glycosylated with a carbohydrate content ranging from 16% in the lightest glycoform to 28% in the heaviest one. As shown in the MALDI spectrum of a glycopeptide arising from the tryptic digest of PG, carbohydrate microheterogeneity gives rise to multiple peaks with mass differences corresponding to the carbohydrate residues (Figure 1). Up to 14 Man residues are present in the high-mannose type glycan chain

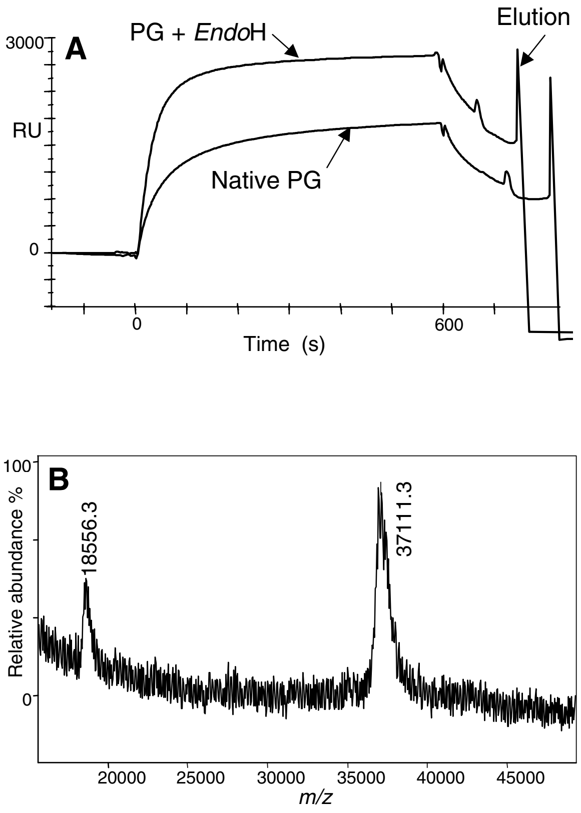

**Figure 2. A**. Sensorgrams of the interaction between the immobilised PGIP-2 and either native *Fm*PG or *Fm*PG deglycosylated with EndoH. **B**. The deglycosylated *Fm*PG retained on the sensor surface is eluted and analysed by MALDI-TOF-MS. The difference between the observed mass ($M_{av}$ = 37110 Da) and the mass calculated for the polypeptide (36198 Da) is in good agreement with the presence of four GlcNAc residues attached at the four potential *N*-glycosylation sites. The peak at *m/z* 18556 corresponds to the doubly charged ion of the protein

Copyright © 2001 John Wiley & Sons, Ltd.

typical of the yeast-secreted proteins. To investigate whether the glycosylation of the enzyme is involved in the binding of *Fm*PG to PGIP-2, PG deglycosylation was performed using endo-H, which leaves a single GlcNAc residue present at each of the occupied Asn residues. The enzymatic deglycosylation does not affect the proper folding of the enzyme which maintains activity and produces an oligogalacturonide profile comparable to that produced by the wild type enzyme (Bergmann *et al.*, 1996). Native PG and PG treated with endo-H were passed over a sensor chip with immobilised PGIP-2 (Figure 2). The sensorgram in Figure 2**A** shows that the deglycosylated enzyme binds to the inhibitor. Interestingly, the affinity for the deglycosylated enzyme is higher than that of the glycosylated form, with a difference in the equilibrium response of ca. 1000 RU. We concluded that not only the glycosylation of *Fm*PG is not required for binding to PGIP, but also that the presence of the glycans might sterically reduce the number of contacts between the two proteins.

A modification of the method developed by Sönksen *et al.*, (1998) was used for the recovery of the affinity-bound enzyme from the sensor surface for mass spectrometric analysis (Figure 2**B**). The amount of protein eluted from the sensor chip was calculated to be ca. 40 fmol, as based on the molecular weight of PG. The protein eluted from the sensor surface showed an average molecular mass of 37110 Da, in good agreement with the mass calculated for the polypeptide (36198 Da) plus four GlcNAc residues attached at the four potential *N*-glycosylation sites.

In order to locate the domain of PG recognised by the inhibitor, a peptide mixture was prepared by limited proteolysis of the native enzyme with Endoproteinase Lys-C (Figure 3). The peptide mixture derived by treatment with Endoproteinase Lys-C was passed in flow over the sensor surface with immobilised PGIP-2, and over a reference flow-cell as a control. One peptide with *m/z* 6479.9 was specifically recovered from the flow-cell with immobilised PGIP-2 (Figure 3**B**). In addition to the

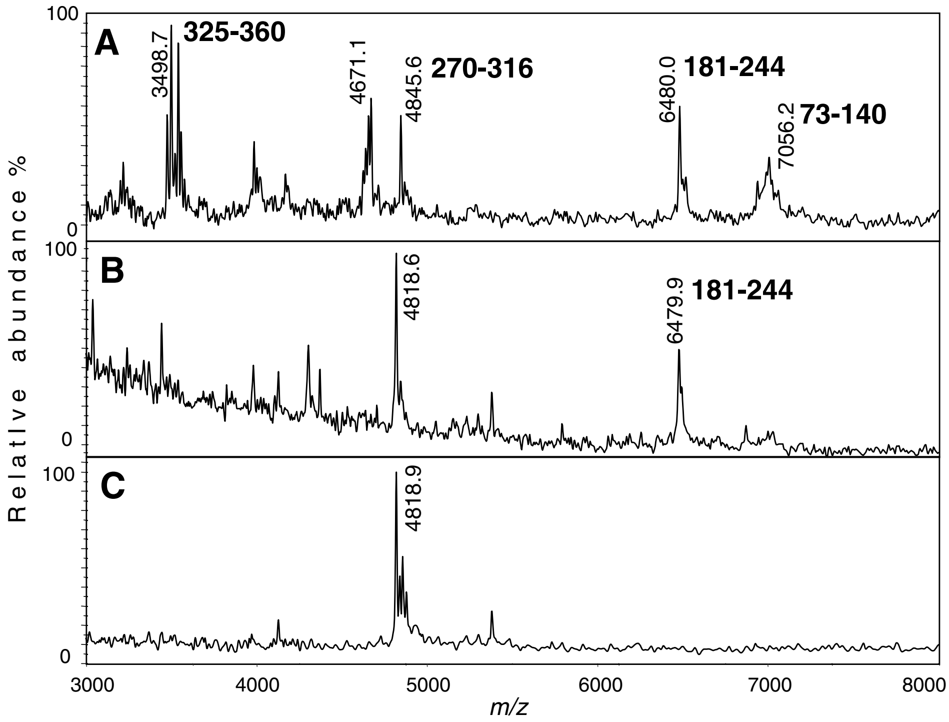

**Figure 3. A.** MALDI spectrum of the peptide mixture obtained by the digestion of native *Fm*PG with endoproteinase LysC. The mixture was injected in the BIACORE and passed over a sensor chip with immobilised PGIP and over a reference flow-cell as a control; **B.** MALDI spectrum of the sample eluted from the PGIP surface. The ion at *m/z* 6479.9 corresponds to the peptide comprising the residues 181–244; **C.** MALDI spectrum of the sample eluted from the reference flow-cell. The ion at *m/z* 4818.6, present also in spectrum B, is a contaminant frequently observed after elution

Copyright © 2001 John Wiley & Sons, Ltd.

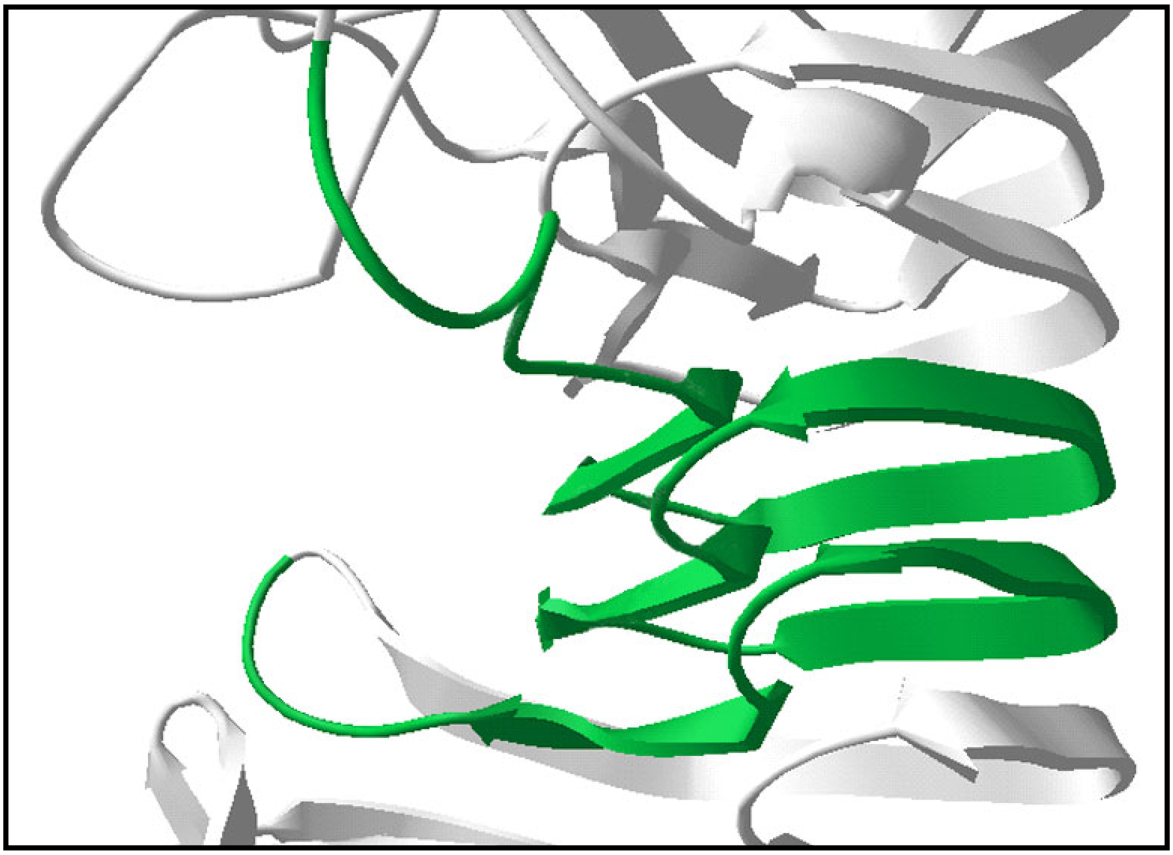

**Figure 4.** Close up of the structure of the peptide 181–144 (shown in green) inside the active site cleft of *Fm*PG

peak at *m*/*z* 6479.9, a non-specific peak at *m*/*z* 4818.6, also present in the fraction eluted from the reference flow-cell, was detected (Figure 3**B** and **C**). The last peak is not present in the original mixture of peptides (Figure 3**A**) and is probably a contaminant that comes from the elution procedure. The peak at m/z 6479.9 corresponds to the PG fragment spanning from amino acid 181 to amino acid 244 comprising several residues of *Fm*PG which are conserved in all the known fungal PGs and form the active site of this class of enzymes (Armand *et al*., 2000). Among them Asp191, Asp212, Asp213 correspond to residues that in PGII from *Aspergillus niger* (*An*PGII) have been shown to be involved in catalysis, and His 234 corresponds to a residue that in *An*PGII is thought to play an indirect role in catalysis (Armand *et al*., 2000). Recently we reported the crystal structure of *Fm*PG, that allows us to locate the peptide 181–244 within the putative active site cleft of the enzyme (Figure 4). By site-directed mutagenesis and SPR analysis, we have demonstrated that several amino acids of the active site and His188, at the edge of the active site cleft, are critical for the formation of the complex with PGIP (Federici *et al*., 2001). Our isolation of the peptide 181–244 which has a strong capacity of interaction with PGIP-2, confirms that most of the residues critical for the interaction are located within this region.

### Acknowledgement

This research was supported by the European Community Grant QLK3–CT99–089, the Institute Pasteur-Fondazione Cenci Bolognetti and the Giovanni Armenise-Harvard Foundation.

### References

Armand S, Wagemaker MJ, Sanchez-Torres P, *et al*. 2000. The active site topology of *Aspergillus niger* endopolygalacturonase

Copyright © 2001 John Wiley & Sons, Ltd. 

II as studied by site-directed mutagenesis. *J Biol Chem* **275**: 691–696.

Bergmann CW, Cook B, Darvill AG, Albersheim P, Bellincampi D, Caprari C. 1996. The effect of glycosylation of *endo*polygalacturonases and polygalacturonase inhibiting proteins on the production of oligogalacturonides. In *Pectins and Pectinases*, Visser J, Voragen AGJ. (eds). Elsevier Science: Amsterdam, The Netherlands; 275–282.

Caprari C, Mattei B, Basile ML, *et al.* 1996. Mutagenesis of endopolygalacturonase from *Fusarium moniliforme*: Histidine residue 234 is critical for enzymatic and macerating activities and not for binding to polygalacturonase-inhibiting protein (PGIP). *Mol Plant-Microbe Interact* **9**: 617–624.

Caprari C, Richter A, Bergmann C, *et al.* 1993. Cloning and characterization of the gene encoding the *endo*polygalacturonase of *Fusarium moniliforme*. *Mycol Res* **97**: 497–505.

Cervone F, Castoria R, Leckie F, De Lorenzo G. 1997. Perception of fungal elicitors and signal transduction. In *Signal Transduction in Plants*, Aducci P. (ed). Birkäuser Verlag: Basel/Switzerland; 153–177.

Federici L, Caprari C, Mattei B, *et al.* 2001. Structural requirements of *endo* polygalacturonase for the interaction with PGIP (polygalacturonase inhibiting protein). *PNAS* in press.

Gobom J, Nordhoff E, Mirgorodskaya E, Ekman R, Roepstorff P. 1999. Sample purification and preparation technique based on nano-scale reversed-phase columns for the sensitive analysis of complex peptide mixtures by matrix-assisted laser desorption/ionization mass spectrometry. *J Mass Spectrom* **34**: 105–116.

Kobe B, Deisenhofer J. 1996. Mechanism of ribonuclease inhibition by ribonuclease inhibitor protein based on the crystal structure of its complex with ribonuclease A. *J Mol Biol* **264**: 1028–1043.

Leckie F, Mattei B, Capodicasa C, *et al.* 1999. The specificity of polygalacturonase-inhibiting protein (PGIP): a single amino acid substitution in the solvent-exposed β-strand/β-turn region of the leucine-rich repeats (LRRs) confers a new recognition capability. *EMBO J* **18**: 2352–2363.

Mattei B, Bernalda MS, Federici L, Roepstorff P, Cervone F, Boffi A. 2001. Secondary structure and post-translational modifications of the leucine-rich repeat protein PGIP (polygalacturonase-inhibiting protein) from *Phaseolus vulgaris*. *Biochemistry* **40**: 569–576.

Nelson RW, Nedelkov D, Tubbs KA. 2000. Biomolecular interaction analysis mass spectrometry. BIA/MS can detect and characterize proteins in complex biological fluids at the low- to subfemtomole level. *Anal Chem* **72**: 404–411.

Papageorgiou AC, Shapiro R, Acharya KR. 1997. Molecular recognition of human angiogenin by placental ribonuclease inhibitor – an X-ray crystallographic study at 2.0 A resolution. *EMBO J* **16**: 5162–5177.

Sönksen CP, Nordhoff E, Jansson O, Malmqvist M, Roepstorff P. 1998. Combining MALDI mass spectrometry and biomolecular interaction analysis using a biomolecular interaction analysis instrument. *Anal Chem* **70**: 2731–2736.

Williams C, Addona TA. 2000. The integration of SPR biosensors with mass spectrometry: possible applications for proteome analysis. *Trends Biotechnol* **18**: 45–48.

Copyright © 2001 John Wiley & Sons, Ltd. 
